# Evaluation of Reporting Quality in Randomised Controlled Trials of Acupuncture for Acute Herpes Zoster by the CONSORT Statement and STRICTA Guidelines

**DOI:** 10.1155/2020/4308380

**Published:** 2020-02-18

**Authors:** Guifeng Qian, Jingchun Zeng, Liming Lu, Wenya Pei, Kun Liu, Zhenke Luo, Yalin She, Pande Zhang, Guohua Lin

**Affiliations:** ^1^Department of Neurology, Zhujiang Hospital, Southern Medical University, Guangzhou 510282, China; ^2^Department of Rehabilitation, The First People's Hospital of Foshan (Affiliated Foshan Hospital of Sun Yat-sen University), Foshan 528000, China; ^3^Department of Acupuncture, The First Affiliated Hospital, Guangzhou University of Chinese Medicine, Guangzhou 510405, China; ^4^Clinical Research and Data Center, Medical College of Acu-Moxi and Rehabilitation, Guangzhou University of Chinese Medicine, Guangzhou 510405, China; ^5^The First Affiliated Hospital, Sun Yat-Sen University, Guangzhou 510080, China; ^6^The First Clinical Medical College, Guangzhou University of Chinese Medicine, Guangzhou 510405, China

## Abstract

**Objective:**

To explore the methods for improving the reporting quality of randomised controlled trials (RCTs) on acupuncture through evaluating the reporting quality in RCTs of acupuncture for acute herpes zoster by the CONSORT statement and STRICTA guidelines.

**Methods:**

English and Chinese databases were searched from database creation until October 2018 and updated to July 2019. The basic characteristics and methodological quality of the literatures included were evaluated based on the CONSORT statement and the STRICTA guidelines. Descriptive statistical analysis was used in this study. The agreement between the two researchers of all items was calculated by Cohen's kappa statistics.

**Results:**

A total of 40 RCTs were included. Based on the CONSORT statement, items “Background,” “Randomised” in the title or abstract,” “Statistical methods,” and “Outcomes and estimation” were good reporting, with positive rates >80%. However, the quality of reporting in items “Sample size,” “Allocation concealment,” “Implementation,” “Blinding,” “Flow chart,” “Intent-to-treat analysis,” “Ancillary analyses,” and “Clinical Trials Register” was very poor, with positive rates <10%. Based on the STRICTA guidelines, good reporting existed in items “Acupuncture rational,” “Points used,” “Needle stimulation,” “Needle retention time,” “Course of treatment,” “Control intervention,” and “Treatment frequency,” with positive ratings >80%. The reporting quality of items “Numbers of needles inserted,” “Depth of insertion,” “Responses elicited,” and “Practitioner background” was lower, with positive rates <50%. The agreement of most items was judged as moderate, substantial, or good.

**Conclusion:**

The reporting quality of RCTs in acupuncture for acute HZ is generally inadequate. It is necessary that researchers and journal editors learn and raise the adoption of the CONSORT statement and STRICTA guidelines to enhance the reporting quality of the RCTs in acupuncture.

## 1. Introduction

Herpes zoster (HZ) is caused by activation of varicella-zoster virus resulting in sensory ganglia infection [1]. The major symptoms of acute HZ are the severe pain, characteristic rash, and distinctive distribution, which reduce quality of life [2]. HZ can occur in all age groups especially in elderly. The incidence rate of HZ rises sharply from the age of 60 and reaches 11 cases per 1000 at the age of 90, roughly half of those who reach 85 years old will develop HZ [3]. The standard treatment for HZ is antiviral therapy, despite the progress of the vaccine advanced. Research has shown that the effect of HZ vaccine lasted only 8 years [4], so adults vaccinated before the age of 60 years might not be protected at the highest risk of HZ. The efficacy of the vaccine still needs further research and improvement [5]. More and more physicians and patients have accepted acupuncture as a complement therapy for a series of diseases especially pain in western countries. A cross-sectional study investigated that the pain was the top acupuncture indications and was nearly half of the clinical problem acupuncturists encountered in the US clinics [6]. Acupuncture was reported for the treatment of HZ [7–10], which showed that it relieves pain of HZ, shortens the healing of the rash, and reduces the occurrence of postherpetic neuralgia (PHN). But no studies have evaluated the reporting quality of these papers.

The “Consolidated Standards of Reporting Trials” (CONSORT) guidelines, updated in 2001 and 2010, provided checklists for the author to write papers for publication [11, 12]. It is widely used to evaluate the reporting quality of clinical trials today. The “Standards for Reporting Interventions in Controlled Trials of Acupuncture” (STRICTA) guidelines, extending the CONSORT guidelines, is to improve the reporting quality of clinical trials for acupuncture [13, 14]. A series of studies showed they help readers obtain information about research design, intervention implementation, data analysis, and so on [15–18]. They have positively impacted on reporting quality of the trials. Based on the above guidelines, this study was conducted to clarify the reporting quality of acupuncture for acute HZ by CONSORT statement and STRICTA guidelines.

## 2. Methods

### 2.1. Search Strategy

The databases Ovid Embase, Ovid Medline, Cochrane Library, WanFang database, Chongqing Weipu (VIP), and China National Knowledge Infrastructure (CNKI) were searched from creation until October 2018 and updated it to July 2019. The following search terms were used in Chinese and English: (Acupuncture OR Acupuncture therapy OR Electro-acupuncture OR Auricular acupuncture OR Scalp acupuncture OR Fire needle OR Warming needle OR Needle OR Acupuncture point OR Meridian OR Acupoint OR Jingluo) AND (Herpes zoster OR Shingles OR Varicella-zoster virus). All articles were published either in Chinese or English. The details of the search strategy can be found in Supplementary [Supplementary-material supplementary-material-1].

### 2.2. Inclusion and Exclusion Criteria

#### 2.2.1. Types of Studies

We included any clinical reports assessing the efficacy of acupuncture in the treatment of acute HZ and mentioning “Randomised Controlled Trials” in the title, abstract, or text, regardless of blinding, implementation, or allocation concealment were used or not in the study. Nonrandomized papers, review, abstract, and case report were included. Any studies with no available data or outcomes for extraction have been excluded.

#### 2.2.2. Types of Participants

Patients of any gender, age, or ethnicity with HZ in the acute phase were eligible. Herpes zoster is diagnosed clinically by the pain, characteristic rash, and distinctive distribution [19]. The acute phase of HZ usually occurs during the 30 days after the onset of the characteristic rash [20]. Patients of any types of HZ with the exception of visceral HZ, meningeal HZ, and PHN were qualified.

#### 2.2.3. Types of Interventions

Different forms of acupuncture techniques or needle were included consisting of manual acupuncture, electroacupuncture, fire or warm needle, scalp acupuncture, and auricular acupuncture, except laser acupuncture and acupressure. The intervention of the experimental group was acupuncture alone or acupuncture combined with medication same as the control group. The control group used placebo, no treatment, antivirals, neurotrophic drugs, corticosteroids, and analgesics, while acupuncture, traditional Chinese medicine, proprietary Chinese medicine, moxibustion, cupping, or physical therapy did not contained.

### 2.3. Selection of Reports

Firstly, two researchers (GQ and JZ) reviewed the title and abstract of citations retrieved and read the full report through NoteExpress separately. Any article that does not meet incorporate criteria or match exclusions standards moved into the specified folder with different labels in NoteExpress. For example, nonrandomized controlled trials, case report, animal experiments, comments, and reviews are removed in the specified folder. The entire team discusses any uncertain literature and makes the final decision.

### 2.4. Data Extraction

Two researchers (WP and KL) used the EpiData software to selected information from the final documents after receiving training of research and statistical methods about the CONSORT and STRICTA checklists. The general information includes the title of the paper, the author, the year of publication, the name of the journal, the type of journal, and the language of the publication. Any information unmentioned was encoded as “not reported.” Two researchers analyzed the full text of 40 articles and performed data extraction.

### 2.5. Assessment of Reporting Quality

We assessed the reporting quality of RCTs in acupuncture for acute HZ according to the CONSORT statement and STRICTA guidelines. Items of them can be found in Tables 1 and 2. Every article was reviewed by two investigators (GQ and JZ). If report 0 indicates that the item is not explicitly stated, it is marked as 1. The agreement between the two researchers been evaluated through calculating Cohen's kappa. Agreement was judged as good if *κ* was >0.8, substantial if 0.6 < *κ* ≤ 0.8, moderate if 0.4 *<* *κ* ≤ 0.6, fair if 0.2 *<* *κ* ≤ 0.4, and poor if *κ* was ≤0.2. Any disagreements or difficulties were solved through conversation in the process. 95% CI and Cohen's kappa of each variable were performed by the Statistical Package for the Social Sciences (SPSS) version 20.0.

## 3. Results

**Figure 1:**
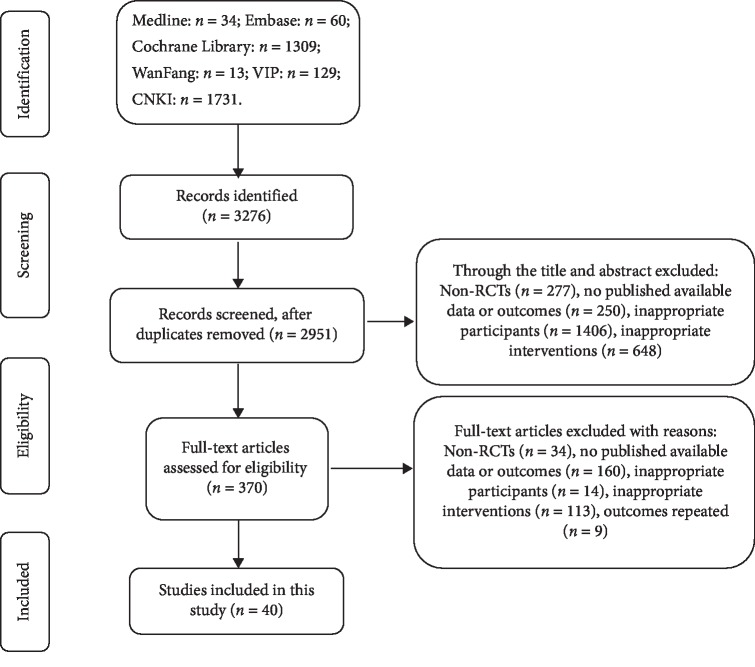
Flow chart of study selection of RCTs of acupuncture for acute HZ.

After searching, 34 Medline, 60 Embase, 1309 Cochrane Library, 13 WanFang, 129 VIP, and 1731 CNKI, totally 3276 recordings were retrieved (Figure 1). Among them, 1403 were English, and 1873 were Chinese. In accordance with the inclusion of the literature, excluding standards, and screening requirements, 40 RCT papers screened at last in Supplementary [Supplementary-material supplementary-material-1].

### 3.1. Characteristics of Included Trials

#### 3.1.1. Year of Publication

**Figure 2:**
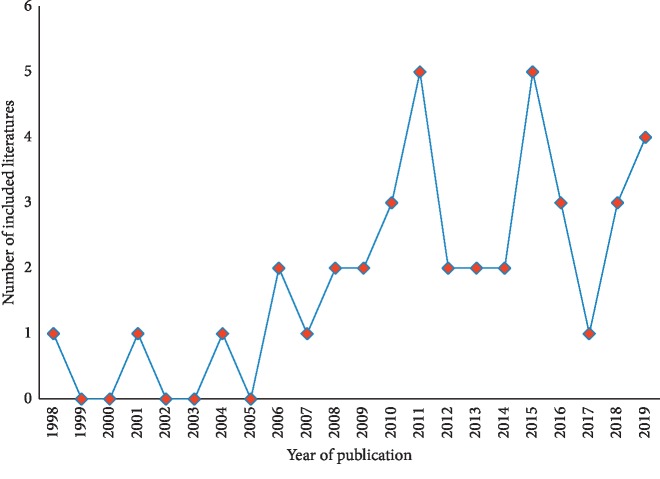
Number of RCTs of acupuncture for acute HZ published per year.

In total, 40 RCTs were mainly published in the last 10 years including 1998 to 2019 (Figure 2). The interventions of them were manual acupuncture (19, 47.50%), electroacupuncture (11, 27.50%), scalp acupuncture (1, 2.50%) and fire needle (9, 22.50%), without ear acupuncture, sham acupuncture, or another intervention.

#### 3.1.2. Journals and Languages

The 40 trials were published in 34 different journals. 11 (27.50%) papers were published in general medical journals, 18 (45.00%) papers in traditional or alternative medicine journals regarding journal category, 10 (25.00%) in specialist medical journals, and 1 (2.50%) paper was master's thesis. 2 (5.00%) were published in Journal of New Chinese Medicine, 1 (2.50%) in Acupuncture Research, and 1 (2.50%) in the Shanghai Journal of Acupuncture Moxibustion. Only 1 paper (2.50%) was published in the Complementary Alternative Medicine in English, and the remaining 39 (97.22%) papers are all published in Chinese.

#### 3.1.3. Funding Sources

Only 8 studies (20.00%) reported their sources of funding. 3(7.50%) received funding from national, 3 (7.50%) from university or hospitals, and 2 (5.00%) from regional, respectively. There is no report which received funding from pharmaceutical companies or research agencies in our sample.

### 3.2. Completeness of Reporting for the CONSORT Items

The data of reporting quality are listed in Table 1 based on the CONSORT statement. Good reporting were items “Background,” “Randomised” in the title or “abstract,” “Statistical methods,” “Outcomes and estimation,” and “Interpretation,” with positive rates >80%. However, the reporting quality in items “Sample size,” “Allocation concealment,” “Implementation,” “Blinding,” “Flow chart,” “Intent-to-treat analysis,” “Ancillary analyses,” “limitation,” and “Clinical Trials Register” was very poor with positive rates <10%.

### 3.3. Completeness of Reporting for the STRICTA Items

The reporting quality ratings for the needling details are listed in Table 2. Based on the STRICTA guidelines, good reporting existed for the items “Acupuncture rational,” “Points used,” “Needle stimulation,” “Needle retention time,” “Course of treatment,” and “Treatment frequency,” with positive ratings >80%. However, the reporting quality of items “Numbers of needles inserted,” “Depth of insertion,” and “Responses elicited” was lower, with positive rates <50%, and “Practitioner background” was poor, with positive rates <10%.

The agreement between two reviewers of all items was observed in this study, and the agreement of mostly items was judged as moderate, substantial or good, except items “Implementation,” “Responses elicited,” and “Needle type” was assessed fair.

## 4. Discussion

This study has showed that the reporting quality of RCTs in acupuncture for HZ was dissatisfied. We need to pay attention as a lot of papers did not strictly comply with CONSORT statement and STRICTA guidelines in this study. Low-quality clinical research influences other researchers to evaluate the study. Accurate and standardised reporting not only reduces the bias of systematic reviews but also contributes to medical decision-making. Acupuncturists can easily grasp effective operational procedures with high-quality reporting.

In the 40 reports, most of them use “random,” “random group,” and other words simply to describe randomisation, and only 13 studies (32.50%) report the method used to generate the random allocation sequence. Adequate randomisation is an effective measure to control the selective offsets and assure the authenticity of the results [21]. The researchers should use the scientific random sequence generation methods and describe it in detail to ensure the feasibility and repeatability of the study.

No literature referred allocation concealment, intent-to-treat analysis, blinding of participants or care providers;only 1 paper (2.50%) showed outcome assessors blinded after assignment to interventions. Research confirmed that allocation concealment, blinding, and intent-to-treat analysis are important protective measures to prevent implementation, measurement bias, and overestimate the effect especially in the evaluation of subjective outcome indicators such as pain, itching, and mood [22–24]. Therefore, these aspects need to be improved in later research.

The sample size estimation can avoid false negative results between intervention and control groups. There were only 2 reports (5.00%) referred to the sample size estimates, and samples of 12 RCTs (30.00%) were more than 100. The lack of correct sample size estimation will lead to the lower test performance of RCT and affect the authenticity and reliability of the research results.

We select STRICTA guidelines to assess the quality of the “intervention” in report, as it has more detailed and specific reporting criteria for acupuncture. According to the STRICTA guidelines, the reporting quality of items “Numbers of needles inserted,” “Depth of insertion,” “Responses elicited,” and “Practitioner background” was lower. A study indicates that the number of needles inserted and course of treatment were associated with outcomes in the treatment of pain [25]. Another study concluded that needling at correct acupoint locations was especially important to the effect of acupuncture for chronic pain [26]. However, there was little evidence to confirm the characteristics of acupuncture or acupuncturists have a wide variation of effects on pain at present, which required large sample sizes trials to evaluate. Therefore, it is significant to report all items of STRICTA guidelines.

Otherwise, there are several limitations in this study. Firstly, we did not search literature published in Japanese or Korean, and we only included trials published in English and Chinese. Secondly, we did not extract all items from the CONSORT 2010 statement and the STRICTA guidelines. Lastly, the literature finally included in this study is mainly in Chinese, and only one paper is in English.

## 5. Conclusions

The study indicates that the quality of reporting in RCTS on acupuncture for HZ was significantly lower and needs substantial improvement. In addition, most editors and researchers in Chinese are not familiar with the CONSORT statement and its extensions [16]. So, we recommend that the authors reporting and designing RCTs to abide by them and journal editors learn and raise the adoption of them to enhance the reporting quality of the RCTs in acupuncture.

## Figures and Tables

**Table 1:** Reporting quality using items from the CONSORT statement (*n* = 40 studies).

Reporting quality using items from the CONSORT statement (*n* = 40 studies)		No. of positive trials	%	95% CI	Cohen's Kappa	95% CI
“Randomised” in the title or abstract	“Randomised” in the title or abstract	35	88	77 to 99	0.77	0.63 to 0.91
Background	Adequate description of the scientific background and explanation of rationale	36	90	80 to 99	0.84	0.72 to 0.96
Trial design	Description of trial design (such as parallel and factorial) including the allocation ratio	7	18	5 to 31	0.81	0.68 to 0.94
Participants	Description of the eligibility criteria for participants	21	53	37 to 69	0.70	0.55 to 0.85
Interventions	Details of the interventions intended for each group(refer Table 2)	—	—	—	—	—
Outcomes	Definition of primary (and secondary when appropriate) outcome measures	31	78	64 to 92	0.59	0.43 to 0.75
Sample size	Description of sample size calculation	2	5	0 to 12	1	—
Randomisation	(a) Method used to generate the random allocation sequence	13	33	18 to 48	0.70	0.55 to 0.85
	(b) Type of randomisation details of any restriction	0	0	—	—	—
Allocation concealment	Description of the method used to implement the random allocation sequence assuring the concealment until interventions were assigned	0	0	—	—	—
Implementation	Who generated the random allocation sequence, who enrolled participants, and who assigned participants to interventions	3	8	0 to 17	0.36	0.20 to 0.51
Blinding	If done, who was blinded after assignment to interventions					
	(a) Participants	0				
	(b) Care providers	0				
	(c) Outcome assessors	1	3	0 to 9	1	—
Statistical methods	Description of the statistical methods used to compare groups for primary outcomes, subgroup analyses, or adjusted analyses	34	85	73 to 97	0.47	0.31 to 0.63
Flow chart	Details on the flow of participants through each stage of the trials	2	5	0 to 12	1	—
Recruitment	Dates defining the periods of recruitment and follow-up	27	68	53 to 83	0.58	0.41 to 0.74
Baseline data	An outline of baseline demographic and clinical characteristics of each group	29	73	58 to 88	0.78	0.64 to 0.92
Intent-to-treat analysis	Number of participants in each group included in each analysis and whether it was done by “intention-to-treat”	0	0	—	1	—
Outcomes and estimation	For each primary and secondary outcome, summary of results for each group was given as well as the estimated effect size and its precision (e.g., 95% CI)	40	100	—	1	—
Ancillary analyses	Clear statement of whether subgroup/adjusted analyses were prespecified or exploratory	0	0	—	1	—
Adverse events	Description of all important adverse events in each group	7	18	5 to 30	0.92	0.83 to 1
Clinical trials register	Whether to conduct clinical trial registration	1	3	0 to 9	1	—
Funding	Fund support	8	20	7 to 33	0.92	0.81 to 1

**Table 2:** Reporting quality of details of needling from STRICTA (*n* = 40).

Details of needling		No. of positive trials	%	95% CI	Cohen's Kappa	95% CI
Acupuncture rational	Reasoning for treatment provided	38	95	87 to 100	0.66	0.50 to 0.81
*Needling details*						
Points used	Names or location of points used	38	95	87 to 100	1	—
Numbers of needles inserted	Number of needle insertions per session	17	43	27 to 59	0.85	0.73 to 0.97
Depth of insertion	Depth of insertion	18	45	29 to 61	0.56	0.40 to 0.72
Responses elicited	Responses sought (e.g., *de qi* or muscle twitch response)	19	48	32 to 64	0.37	0.21 to 0.53
Needle stimulation	Needle stimulation (e.g., manual or electrical)	34	85	73 to 97	0.46	0.30 to 0.62
Needle retention time	Needle retention time per session	38	95	88 to 100	1	—
Needle type	Needle type (diameter, length and manufacturer or material)	27	68	53 to 83	0.38	0.22 to 0.54
*Treatment regimen*						
Treatment frequency	Treatment frequency	35	88	77 to 98	0.86	0.75 to 0.97
Course of treatment	Treatment course	35	88	77 to 98	0.77	0.63 to 0.90
Practitioner background	Description of acupuncturists	1	3	0 to 9	1	—
Control intervention	Precise description of the control or comparator.	40	100	—	1	—

## Data Availability

The data used to support the findings of this study are included within the article and supplementary information.

## Supplementary Materials

Supplementary Materials The main features of the 40 included studies and their references in this paper.
