# Supplementary material for: Evaluation of Reporting Quality in Randomised Controlled Trials of Acupuncture for Acute Herpes Zoster by the CONSORT Statement and STRICTA Guidelines
Source: Evid Based Complement Alternat Med. 2020 Feb 18;2020:4308380. doi: 10.1155/2020/4308380 (PMC7049440; doi:10.1155/2020/4308380)
Supplement: Supplementary Materials — The main features of the 40 included studies and their references in this paper. [file 4308380.f1.pdf]

Characteristics of 40 included studies

No.	Included studies	Year	No. of patients	Language	Acupuncture of Treatment group	Course (days)	Duration (days)
1	Xiu jH1998[1]	1998	40	Chinese	Manual acupuncture	1 to 7	7 to 21
2	Xia ZX2001[2]	2001	161	Chinese	Electro-acupuncture	1 to 3	10
3	Li JX2004[3]	2004	56	Chinese	Manual acupuncture	1 to 10	10
4	Li JW2006[4]	2006	191	Chinese	Scalp acupuncture	1 to 10	1 to 10
5	Wang SL2006[5]	2006	120	Chinese	Manual acupuncture	1 to 10	15
6	Lin Y2007[6]	2007	121	Chinese	Manual acupuncture	1 to 7	10
7	Yue J2008[7]	2008	68	Chinese	Fire needle	1 to 5	14
8	Wan LQ2008[8]	2008	156	Chinese	Manual acupuncture	3 to 7	7 to 14
9	Zhang P2009[9]	2009	87	Chinese	Electro-acupuncture	4 to 14	14
10	Li X2009[10]	2009	80	Chinese	Electro-acupuncture	1 to 7	10
11	Tong XY2010[11]	2010	60	Chinese	Electro-acupuncture	1 to 3	30 to 60
12	Chen LY2010[12]	2010	92	Chinese	Manual acupuncture	2 to 15	10
13	Lin LL2010[13]	2010	78	Chinese	Manual acupuncture	1 to 7	Not mentioned
14	Zhang AZ2011[14]	2011	46	Chinese	Electro-acupuncture	1 to 7	21
15	Li LX2011[15]	2011	58	Chinese	Electro-acupuncture	1 to 7	10
16	Lin QQ[16]	2011	160	Chinese	Electro-acupuncture	1 to 6	14
17	Zheng Y[17]	2011	67	Chinese	Manual acupuncture	1 to 7	5
18	Ursini T2011[18]	2011	102	English	Manual acupuncture	1 to 30	28
19	Zhang AZ2012[19]	2012	100	Chinese	Electro-acupuncture	1 to 7	7
20	Zhang AZ2012[20]	2012	100	Chinese	Electro-acupuncture	1 to 7	7 to 28
21	Chen JS2013[21]	2013	25	Chinese	Manual acupuncture	1 to 3	10 to 15
22	Fan YQ2013[22]	2013	103	Chinese	Manual acupuncture	1 to 3	30
23	Chen X2014[23]	2014	88	Chinese	Fire needle	1 to 10	10
24	Zhou TH2014[24]	2014	72	Chinese	Manual acupuncture	1 to 30	10 to 15
25	Sun YZ2015[25]	2015	90	Chinese	Electro-acupuncture	1 to 5	10
26	Liu Y2015[26]	2015	70	Chinese	Fire needle	1 to 7	Not mentioned
27	Zhao D2015[27]	2015	94	Chinese	Manual acupuncture	1 to 13	5 to 15
28	Xu XZ2015[28]	2015	40	Chinese	Manual acupuncture	1 to 2	10
29	Liu J2015[29]	2015	112	Chinese	Manual acupuncture	1 to 5	Not mentioned
30	Yu GH2016[30]	2016	44	Chinese	Fire needle	5 to 18	14
31	Wang XL2016[31]	2016	86	Chinese	Fire needle	1 to 7	10

32	Wang Y2016[32]	2016	78	Chinese	Manual acupuncture	1 to 7	7 to 12
33	Chen ZX2017[33]	2017	72	Chinese	Electro-acupuncture	1 to 7	10
34	Qi HC2018[34]	2018	80	Chinese	Fire needle	1 to 7	10
35	Wei XY2018[35]	2018	90	Chinese	Fire needle	1 to 30	14
36	Jin FP2018[36]	2018	90	Chinese	Manual acupuncture	1 to 7	14
37	Hu B2019[37]	2019	126	Chinese	Fire needle	1 to 30	10
38	Deng SQ2019[38]	2019	60	Chinese	Fire needle	1 to 7	10
39	Zhu XX2019[39]	2019	80	Chinese	Manual acupuncture	1 to 7	15
40	Huang JS2019[40]	2019	80	Chinese	Manual acupuncture	1 to 7	14

References:

[1] X. J. Han, X. Y. Sun, "Treatment of herpes zoster with combination of Chinese and Western medicine", *Medical research communication*, vol., no. 07,pp.27-8,1998.

[2] Z. X. Xia, "81 cases of herpes zoster treated by encircling acupuncture", *Journal of Nanjing University of traditional Chinese medicine*, vol., no. 02,pp.117,2001.

[3] J. X. Li, "Clinical observation of 32 cases of herpes zoster treated with giant needling method", *Chinese general medicine*, vol., no. 09,pp.611,2004.

[4] J. W. Li, "Clinical Observation on the treatment of herpes zoster by scalp acupuncture", *Clinical Journal of acupuncture*, vol., no. 01,pp.32-3,2006.

[5] S. L. Wang, "Observation on the therapeutic effect of 40 cases of herpes zoster treated by five tigers and sheep acupuncture combined with medicine", *New Chinese medicine*, vol., no. 02,pp.62-3,2006.

[6] Y. Lin, X. J. Chen, "Observation on the therapeutic effect of fire acupuncture on 43 cases of herpes zoster", *Fujian Medical Journal*, vol., no. 04,pp.124-5,2007.

[7] J. Yue, Z. Z. Mo, "35 cases of herpes zoster treated by multiple head fire acupuncture", *Fujian traditional Chinese medicine*, vol., no. 01,pp.32-3,2008.

[8] L. Q. Wang, "Acupuncture in the treatment of 78 cases of herpes zoster", *Chinese medicine research*, vol., no. 05,pp.45-6,2008.

[9] P. Zhang, S. Zhang, "Analysis of clinical efficacy of acupuncture in the treatment of herpes zoster cases", *Journal of Baotou Medical College*, vol. 25, no. 06,pp.66-7,2009.

[10] X. Li, H. X. Zhang, G. F. Huang et al., "Randomized controlled observation on the efficacy of electroacupuncture combined with periacupuncture in the treatment of herpes zoster," *Acupuncture research*, vol. 34, no. 02,pp.125-7,2009.

[11] X. Y. Tong, Y. D. Chen, X. P. Wu et al., "Clinical observation of acupuncture combined with western medicine in the treatment of herpes zoster and its effect on substance P in plasma," *Chinese Journal of Dermatology and Venereology of integrated traditional and Western medicine*, vol. 9, no. 04,pp.225-7,2010.

[12] L. Y. Chen, Z. Xu, "Observation on the therapeutic effect of acupuncture on 46 cases of herpes zoster", *Aerospace medicine*, vol. 21, no. 08,pp.1530,2010.

[13] L. L. Lin, "Study on the treatment of herpes zoster," *Chinese medical guidelines*,

vol. 8, no. 35,pp.223-4,2010.
[14] A. Z. Zhang, Q. Zhang, "Effect of Electroacupuncture on cellular immune function in elderly patients with herpes zoster", *Journal of practical Chinese medicine*, vol. 27, no. 07,pp.438-9,2011.
[15] L. X. Li, C. Y. Chen, G. H. Lin et al., "Clinical Observation on 27 cases of acute herpes zoster treated by electroacupuncture," *New Chinese medicine*, vol. 43, no. 03,pp.103-5,2011.
[16] Q. Q. Lin, "Effect analysis of acupuncture combined with acyclovir in the treatment of herpes zoster in the elderly", *Chinese and foreign medical*, vol. 30, no. 35,pp.83-4,2011.
[17] Y. Zheng, M. B. Zhang, L. Jin, "Observation on clinical effect of root cutting therapy combined with syndrome differentiation acupuncture on herpes zoster," *Journal of Liaoning University of traditional Chinese medicine*, vol. 13, no. 05,pp.222-3,2011.
[18] T. Ursini, M. Tontodonati, L. Manzoli et al., "Acupuncture for the treatment of severe acute pain in herpes zoster: results of a nested, open-label, randomized trial in the VZV Pain Study," *BMC Complement Altern Med*, vol. 11, no.,pp.46,2011.
[19] A. Z. Zhang, Y. Liang, "Effect of Electroacupuncture on cellular immune function and early hemorheology in elderly patients with herpes zoster", *Journal of Shandong University of traditional Chinese medicine*, vol. 36, no. 05,pp.407-8,2012.
[20] A. Z. Zhang, Q. Zhang, "Observation on therapeutic effect of electroacupuncture combined with drugs on herpes zoster", *Journal of practical Chinese medicine*, vol. 28, no. 02,pp.122-3,2012.
[21] J. S. Chen, Q. J. Gong, Q. L. Huang et al., "The effect of acupuncture on the expression of c-FLIP in the blood of patients with acute herpes zoster," *Journal of practical medicine*, vol. 29, no. 15,pp.2562-5,2013.
[22] Y. Q. Fan, Z. M. Yang, and J. Gu, "Immune Mechanism Analysis of Chiluo Therapy for Herpes Zoster," *Chinese Medical Guide*, vol. 11, no. 32,pp.502-3,2013.
[23] X. Chen, "Observation on the therapeutic effect of high density fire acupuncture on herpes zoster in 44 cases", *Xinjiang traditional Chinese medicine*, vol. 32, no. 04,pp.49-50,2014.
[24] T. H. Zhou, "Clinical effect and feasibility of acupuncture combined with drugs in the treatment of acute herpes zoster", *Chinese contemporary medicine*, vol. 21, no. 27,pp.127-8,2014.
[25] Y. Z. Sun, L. Li, T. Y. Yu, "Observation on therapeutic effect of Electroacupuncture on herpes zoster", *Shanghai Journal of acupuncture*, vol. 34, no. 11,pp.1046-9,2015.
[26] Y. Liu, L. Han, Y. N. Wang et al., "Observation on the efficacy of fire acupuncture in the treatment of acute herpes zoster," *Electronic Journal of clinical medicine literature*, vol. 2, no. 27,pp.5602-3,2015.
[27] D. Zhao, J. W. Huang, R. L. Huang, "Efficacy analysis of acupuncture combined

with drugs in the treatment of herpes zoster," *Traditional medicine of Asia Pacific*, vol. 11, no. 15,pp.84-5,2015.

[28] X. Z. Xu, Q. M. Wang, "Zheng's" mouse claw acupuncture "in the treatment of 20 cases of herpes zoster of damp heat type," *Journal of external treatment of traditional Chinese Medicine*, vol. 24, no. 06,pp.34-5,2015.

[29] J. Liu, "112 cases of herpes zoster treated by acupuncture combined with western medicine," *Shenzhen Journal of integrated traditional and Western Medicine*, vol. 25, no. 20,pp.34-6,2015.

[30] G. H. Yu, M. H. Ping, X. Guo et al., "Clinical Observation on the treatment of herpes zoster by fire needle pricking," *Guangxi Traditional Chinese Medicine*, vol. 39, no. 03,pp.53-4,2016.

[31] X. L. Wang, H. Z. Chen, Q. F. Zhan et al., "Clinical observation of Lingnan fire needle in the treatment of acute herpes zoster," *Clinical study of traditional Chinese Medicine*, vol. 8, no. 27,pp.118-9,2016.

[32] Y. Wang, "Clinical effect of acupuncture combined with drugs in the treatment of acute herpes zoster and the prevention of sequelae neuralgia," *Chinese prescription drug*, vol. 14, no. 09,pp.96-7,2016.

[33] Z. X. Chen, Y. Xie, Q. Li, "Clinical Observation on the therapeutic effect of modified electroacupuncture on herpes zoster of trunk", *Inner Mongolia traditional Chinese Medicine*, vol. 36, no. 14,pp.102-3,2017.

[34] H. C. Qi, C. L. Zhang, S. P. Li et al., "Observation on the efficacy of fire acupuncture in the treatment of herpes zoster in acute stage," *Clinical study of traditional Chinese Medicine*, vol. 10, no. 20,pp.109-10,2018.

[35] X. Y. Wei, M. H. Zhu, W. Z. Gong et al., "Clinical Observation on 45 cases of herpes zoster treated with fire needle combined with famciclovir dispersible tablets," *Hunan Journal of traditional Chinese Medicine*, vol. 34, no. 11,pp.77-8,2018.

[36] F. P. Jin, "Zheng's cool purgation acupuncture for the treatment of 45 cases of herpes zoster of liver and gall dampness and heat type", *Study of traditional Chinese Medicine*, vol. 31, no. 04,pp.57-9,2018.

[37] B. Hu, "Clinical application of Zan needling with milli fire needle in the treatment of herpes zoster," *Chinese folk therapy*, vol. 27, no. 03,pp.38-9,2019.

[38] S. Q. Deng, X. Li, X. Y. Jian et al., "Clinical efficacy of Lingnan fire needle in the treatment of herpes zoster and its influence on immunity," *Journal of Mathematical Medicine*, vol. 32, no. 05,pp.698-700,2019.

[39] X. X. Zhu. Observation on the therapeutic effect of pressing needle and encircling needling on acute pain of herpes zoster [Master]: *Nanjing University of traditional Chinese Medicine*; 2019.

[40] J. S. Huang, X. Q. Xie, "Zheng's" warming and unblocking acupuncture "in the treatment of 40 cases of herpes zoster of spleen deficiency and dampness accumulation type," *Journal of external treatment of traditional Chinese medicine*, vol. 28, no. 01,pp.46-8,2019.
